# Quantification of mRNA in single cells and modelling of RT-qPCR induced noise

**DOI:** 10.1186/1471-2199-9-63

**Published:** 2008-07-17

**Authors:** Martin Bengtsson, Martin Hemberg, Patrik Rorsman, Anders Ståhlberg

**Affiliations:** 1 Oxford Centre for Diabetes, Endocrinology and Metabolism, University of Oxford, The Churchill Hospital, Oxford, OX3 7LJ, UK; 2 Department of Clinical Sciences, Lund University, Clinical Research Centre, 205 02 Malmö, Sweden; 3 Department of Bioengineering, Imperial College London, South Kensington Campus, London SW7 2AZ, UK; 4 Stem Cell Center, Lund University, BMC B10, 221 84 Lund, Sweden; 5 Department of Ophthalmology and Program in Neurobiology, Children's Hospital Boston, Harvard Medical School, 1 Blackfan Circle, Boston, MA 02115, USA; 6 Department of Clinical Neuroscience and Rehabilitation, Institute of Neurosciences and Physiology, Sahlgrenska Academy at Göteborg University, Medicinaregatan 9A, 413 90 Göteborg, Sweden

## Abstract

**Background:**

Gene expression has a strong stochastic element resulting in highly variable mRNA levels between individual cells, even in a seemingly homogeneous cell population. Access to fundamental information about cellular mechanisms, such as correlated gene expression, motivates measurements of multiple genes in individual cells. Quantitative reverse transcription PCR (RT-qPCR) is the most accessible method which provides sufficiently accurate measurements of mRNA in single cells.

**Results:**

Low concentration of guanidine thiocyanate was used to fully lyse single pancreatic β-cells followed by RT-qPCR without the need for purification. The accuracy of the measurements was determined by a quantitative noise-model of the reverse transcription and PCR. The noise is insignificant for initial copy numbers >100 while at lower copy numbers the noise intrinsic of the PCR increases sharply, eventually obscuring quantitative measurements. Importantly, the model allows us to determine the RT efficiency without using artificial RNA as a standard. The experimental setup was applied on single endocrine cells, where the technical and biological noise levels were determined.

**Conclusion:**

Noise in single-cell RT-qPCR is insignificant compared to biological cell-to-cell variation in mRNA levels for medium and high abundance transcripts. To minimize the technical noise in single-cell RT-qPCR, the mRNA should be analyzed with a single RT reaction, and a single qPCR reaction per gene.

## Background

Cells in a population are in many aspects unique in their characteristics, even in a seemingly homogenous culture or tissue. Single cell gene expression levels–protein as well as mRNA–show large cell-to-cell variations both in a resting state and when exposed to stimuli, stemming in part from the stochastic nature of gene expression [1-6]. This implies that data obtained from a population of cells cannot be assumed to reflect the behaviour of the individual cell. Instead, cells must be assayed one at a time which, in the case of mRNA measurements, requires characterization of femtograms of mRNA [7].

Quantitative reverse transcription polymerase chain reaction (RT-qPCR) offers sufficient sensitivity and it is the gold standard for mRNA quantification [8,9]. Coupled with a method to handle and collect individual cells, quantification of mRNA in single cells is feasible for most laboratories today. Previously published protocols for single-cell RT-PCR are generally non-quantitative and specialized for a particular cell-type or involve laborious purification steps [10-13]. For single-cell mRNA quantification to become common practice, simple and generally applicable methods are needed. Indeed, non-proprietary protocols not requiring purification have been presented, but they involve only weak detergents serving as lysis agents that do not inhibit nucleases [14-19]. Lack of appropriate controls for single-cell measurements makes it difficult to guarantee mRNA integrity during collection and handling of cells.

Noise in RT-qPCR measurements increases with decreasing initial copy numbers [20] and quantification of rare transcripts pushes the measurement to a point where it no longer can be considered quantitative; at best the presence of mRNA will be *detected*. The details of this phenomenon–including the relative contribution of RT and qPCR respectively–are poorly investigated [20,21]. Previous reports utilizing single-cell RT-qPCR have left these questions largely unanswered or with inconsistent results: qPCR induced variation ranging from ~8% (CV) at 4000 cDNA copies [18] down to <1% at two copies [12] have been shown. Without knowledge of the distribution and impact of the technical noise, it is hard to assess the validity of the measurements.

In this study, we present a single-cell RT-qPCR protocol without purification, with maintained mRNA integrity, high reproducibility and no inhibition of RT or PCR reactions. In addition, we investigate the nature of noise in RT-qPCR and through mathematical modelling we determine the accuracy in measurements of single mouse endocrine cells. Our model predicts how the noise in RT and PCR varies with different expression levels, which will aid in planning and design of single-cell RT-qPCR measurements. We conclude that the inherent variability of RT-qPCR is negligible compared to naturally occurring variation between cells for abundantly expressed genes, while uncertainties in the PCR may obfuscate measurements for rare transcripts.

## Results

### Derivation of a purification-free protocol

**Figure 1:**
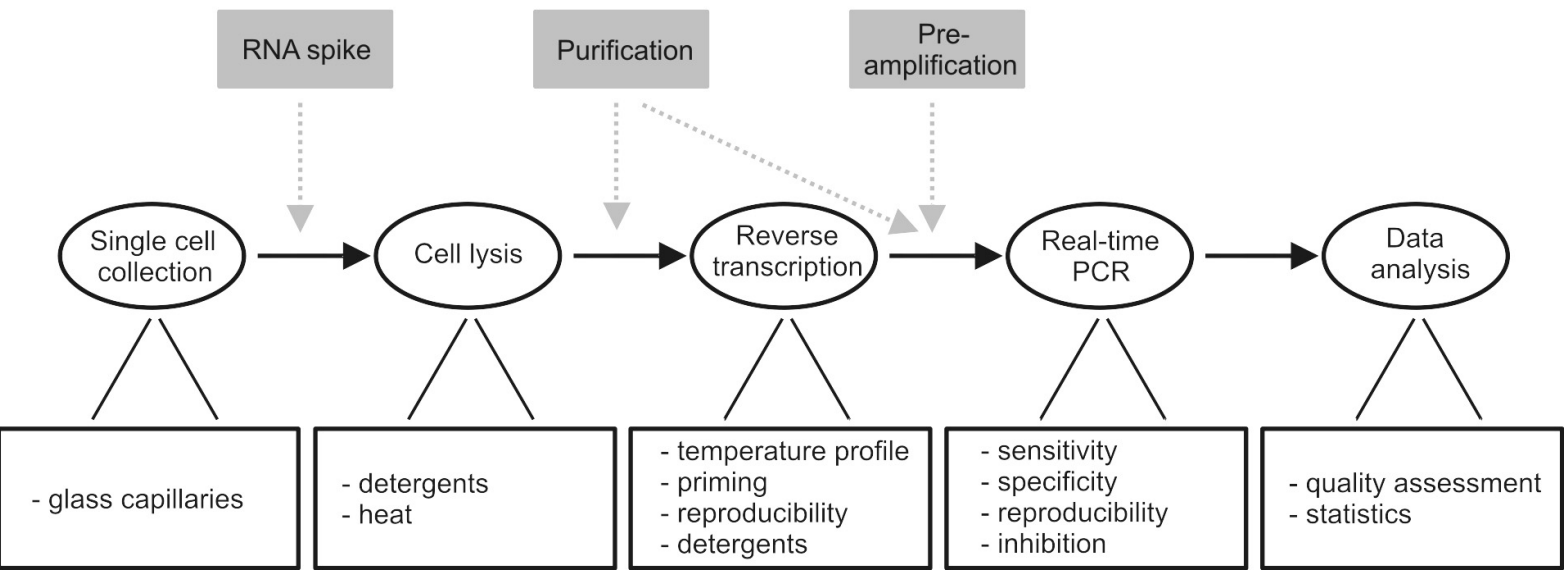
Overview of single-cell gene expression profiling using RT-qPCR.

**Figure 2:**
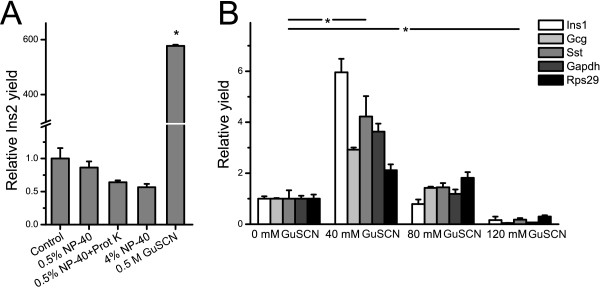
**Evaluation of lysis buffers.** (A) Determination of lysis efficiency. Each bar indicate relative yield of *Ins2 *using a single pancreatic islet (~1000 cells) as starting material. Each islet was treated with indicated concentrations of either NP-40, with and without proteinase K (Prot K) treatment, or guanidine thiocyanate (GuSCN). Only lysis with 0.5 M GuSCN had a significant effect (>500-fold increase) compared to the water control (p < 0.001, n = 3). The value of the control was arbitrarily set to 1. Similar results were obtained for *Gcg *and *Rps29*. (B) Effect of lysis buffers on RT reaction yield. Identical amounts of purified islet total RNA was used as starting material. Relative yields of five genes were analysed: *Ins1*, *Gcg*, *Sst*, *Gapdh *and *Rps29*. Increasing concentrations of GuSCN was added to the RT reaction. There is a significant difference for all genes between control and both 40 mM and 120 mM (p < 0.05) but not 80 mM. The expression value was arbitrarily set to 1 for all genes at 0 mM GuSCN. Values are mean ± SEM for three separate experiments. The experiments in (A) and (B) were carried out without the presence of RNase inhibitor.

The process of single-cell RT-qPCR is summarized in Figure 1. Our approach, which does not involve mRNA purification, requires a lysis solution that is compatible with downstream enzymatic reactions. In addition, it should 1) disrupt the cell membrane; 2) make the mRNA accessible for reverse transcription; and 3) maintain mRNA integrity. Two agents were evaluated for this task: NP-40, a weak, non-chaotropic detergent (also referred to as Igepal CA-630) and guanidine thiocyanate (GuSCN) a strong, chaotropic compound. Five different lysis conditions were tested in terms of their ability to lyse one cell cluster (pancreatic islet of Langerhans, each containing ~1000 cells), assessed by the amount of accessible insulin II (*Ins2*) transcripts using RT-qPCR (Figure 2A). NP-40 had no effect compared to control (water) when used at concentrations of 0.5% or 4%, indicating that these lysis conditions are too weak to dissociate the islet and lyse the cells. Proteinase K is commonly used in lysis protocols, but had no beneficial effect when added in the presence of 0.5% NP-40. GuSCN based lysis buffer provided efficient lysis of the islet using a concentration of 0.5 M and increased the RNA yield 600-fold compared to control conditions (Figure 2A). Lysis of a compact cluster of cells is clearly more challenging than dissociated cells. We evaluated the latter using another cell type, primary astrocytes, and the RNA yield was here within a 2.5-fold range for both 0.5 M GuSCN and 0.5% NP-40 compared to the water control (Additional file 1, Figure 1). This indicates that any of the tested lysis conditions will suffice for complete cell lysis of dissociated cells, while 0.5 M GuSCN is required to break apart cell clusters. In a sample containing a *single *lysed cell, the RNA is assumed to be evenly distributed in the solution. We tested this hypothesis by splitting vortexed single-cell lysates into three separate RT-reactions, followed by qPCR using *Ins2 *primers. Samples lysed in 0.5 M GuSCN showed 80% lower intra-assay variation than cells emptied in 0.5% NP-40, suggesting that GuSCN lyses the cell and efficiently homogenates the mRNA (Additional file 1: Figure 2A).

The presence of potential inhibitors and RNases from the tissue or culture medium may reduce the cDNA yield. We addressed this concern by adding a known amount of purified total islet RNA to tubes containing one of the following: 1) extracellular solution (see the Methods section for details); 2) one cell in 0.5 M GuSCN in water; or 3) ten cells in 0.5 M GuSCN in water (Additional file 1: Table 1). The amount of RNA in the cells was negligible compared to the externally added RNA. The yield was unaffected by the extracellular solution and in fact increased in the presence of cells in GuSCN, a phenomenon investigated further (see below). As an additional control for inhibitors, we added an equal amount (~2000 copies) of in vitro transcribed artificial RNA, based on the cyclophilin E (*Ppie*) gene, to the lysis solution. *Ppie *was measured in every single-cell sample and deviation >1% led to exclusion from further analysis (data not shown).

In Figure 2B, an equal amount of purified RNA was reverse transcribed in the presence of 0, 40, 80 and 120 mM GuSCN, followed by qPCR using five different primer pairs. The RT-reaction efficiency was significantly improved (2- to 6-fold) for all tested genes (*Ins1*, *Gcg*, *Sst*, *Gapdh *and *Rps29*) using 40 mM GuSCN. By contrast, 80 mM GuSCN had no effect whereas 120 mM GuSCN was strongly inhibitory. We also evaluated the addition of 0.5–1.5% 2-mercapto ethanol, but no effect on the RT yield, either alone or in concert with GuSCN, was observed (data not shown). Formation of correct PCR-products was confirmed by agarose gel electrophoresis. Neither of the other two lysis conditions, NP-40 or proteinase K, had any discernible effect on RT yield (Additional file 1: Figure 2B ). Taken together, we chose to use ~0.5 M GuSCN in the single-cell lysis solution due to superior cell lysis ability and positive effect on the RT reaction at the concentration of 40 mM.

### RT- and qPCR-induced noise

The total technical noise level of RT-qPCR was estimated by diluting purified total RNA from four to six different concentrations (equivalent of ~5-5·10^3 ^copies of chromogranin B (*Chgb*), ~10-10^4 ^copies of ribosomal protein S29 (*Rps29*), ~50-10^3 ^copies of SRY-box containing gene 2 (*Sox2*), nestin (*Nes*) and glial fibrillary acidic protein (*Gfap*) and ~500-5·10^5 ^copies of *Ins2 *transcripts). For each dilution, 24 identical RT-qPCR reactions were run. The mRNA concentrations were chosen to primarily cover low-medium abundance transcripts, i.e. *Chgb, Gfap, Nes, Rps29 and Sox2*. Consequently, the concentration of the high abundance *Ins2 *transcript is 50–100 times higher. The qPCR-specific noise was calculated from 30 qPCR replicates (*Rps29*) at five different concentrations of mouse cDNA. The measurements were combined to create a model of RT- and qPCR-induced noise, as well as errors introduced by the liquid handling. The technical reproducibility of RT and qPCR is represented here by the noise strength (η^2 ^= SD^2^/mean^2^), for different initial copy numbers. Assuming that the noise from each reaction is additive, we can write the total noise as η^2^_Total _= η^2^_Dilution _+ η^2^_RT_+ η^2^_PCR _and by fitting this model to the data, we can quantify the contribution of each component. η^2^_Dilution _corresponds to Poisson-type noise which arises when a sample with a low number of molecules is imperfectly diluted. Additional noise is introduced by the RT-reaction and the final term corresponds to the noise introduced by the qPCR step. In Additional file 2, we derive functional forms for each component and we show how they can be assessed from replicate measurements.

**Figure 3:**
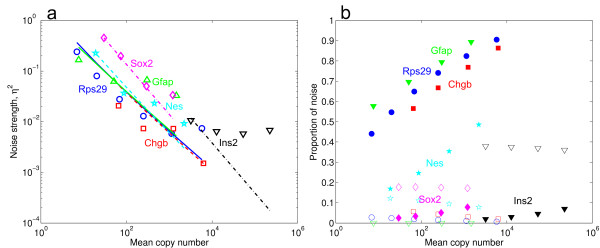
**Noise levels for the measurements of known quantities of mRNA from *Rps29, Chgb, Ins2, Gfap, Nes *and *Sox2*.** (a) The measured noise strength (η^2 ^= SD^2^/mean^2^) for *Rps29 *(blue circles), *Chgb *(red squares) *Ins2 *(black triangles), *Gfap *(green triangles), *Nes *(cyan stars) and *Sox2 *(magenta diamonds) with corresponding fits (lines) obtained using non-linear regression for the mathematical model presented in Additional file 2. The model estimates the RT-efficiency and the results are reasonable for five of the six genes. For *Ins2 *the efficiency is much lower than expected. The poor curve fit for the *Ins2 *gene results from the fact that the *Ins2 *data was generated for much higher copy numbers whereas our model for the PCR-noise was adapted for the low abundances of tge five other genes. (b) The proportion of the total noise for the PCR (filled symbols) and RT reactions (open symbols). Circles, squares and triangles are designated as in (a). The PCR and RT components do not have to add up to 1; the noise stemming from the dilution corresponds to the remaining noise. For *Rps29, Chgb and Gfap*, the PCR noise clearly dominates for all concentrations. For *Ins2 *and *Sox2*, the estimated RT efficiency is very low which means that this reaction will add a larger contribution to the total noise. *Nes *has an intermediate efficiency and for low copy numbers the RT noise dominates but it becomes smaller than the qPCR nosie when more transcripts are analyzed. Furthermore, the copy numbers are relatively high for *Ins2 *which deflates the PCR noise.

Figure 3 shows that the total technical noise is very low at initial mRNA copy numbers down to ~100. The noise model allows us to estimate the RT-reaction efficiency (i.e. the fraction between the resulting number of cDNA copies and the initial number of mRNA copies) for each gene by non-linear regression. We find that the RT-reaction efficiency is 99%, 85%, 67%, 28%, 8% and 3% for *Gfap, Rps29, ChgB, Nes, Sox2 *and *Ins2*, respectively. The estimate for *Ins2 *is clearly much lower than what we would expect and we believe that this is due to insufficient data on the qPCR noise for high cDNA copy numbers for the fitting procedure (see Additional file 2). We conclude that for high RT-efficiencies, the noise originating from the RT reaction is comparatively low, 5–20% of total noise, while the qPCR noise dominates (30–90%) at low concentrations. If the RT-efficiency is low (<10%, i.e. *Sox2 *and *Ins2*), the qPCR noise is insignificant for all concentrations.

### Single-cell RT-qPCR

**Figure 4:**
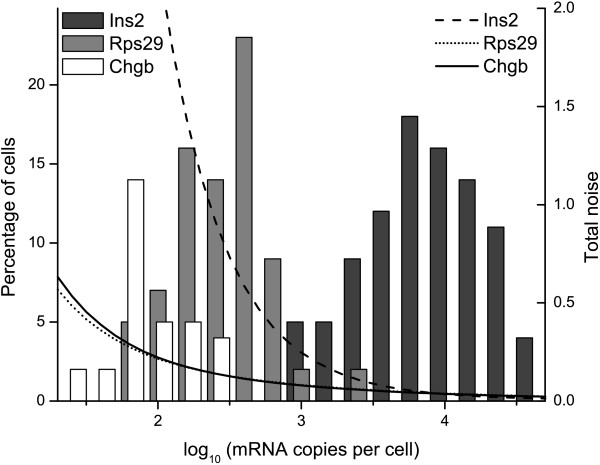
**Histogram showing expression levels of three genes (*Rps29, Chgb *and *Ins2*) in 101 β-cells.** Overlaid curves show the total technical noise levels for RT-qPCR measurements. The biological noise, which can be interpreted as normalized width of each histogram, is 1.0, 0.33 and 1.4 for *Rps29, Chgb *and *Ins2 *respectively.

Using a glass capillary mounted on a micromanipulator, we collected pancreatic β-cells incubated in 3, 6, 10 and 20 mM glucose and emptied the intact cell in 1 μl 0.5 M GuSCN. Following reverse transcription, we subjected each single-cell's cDNA to qPCR using primers for *Rps29, Chgb *and *Ins2 *(Figure 4). The median expression level across the different glucose concentrations for these three genes were 9900, 230 and 110 mRNA copies per cell respectively. Some cells failed to generate a signal (34, 59 and 2% for *Rps29, Chgb *and *Ins2 *respectively). Transcript level heterogeneity is high and the distribution is skewed, which is in agreement with previous studies of eukaryotic cells [5,14,19]. One consequence of this heterogeneity is that the top 10% of the cells with the highest *Ins2 *expression account for 50% of the total *Ins2 *mRNA. Increasing glucose concentrations altered the median *Ins2 *expression from 9000 mRNA copies at 3 mM glucose to 8500, 10000, and 13000 mRNA copies for 6, 10 and 20 mM glucose, respectively (a detailed description is found in Additional file 1: Figure 4). The increase was mainly due to an increasing fraction of cells with very high expression levels. Glucose did not have an effect on *Rps29 *or *Chgb*.

**Figure 5:**
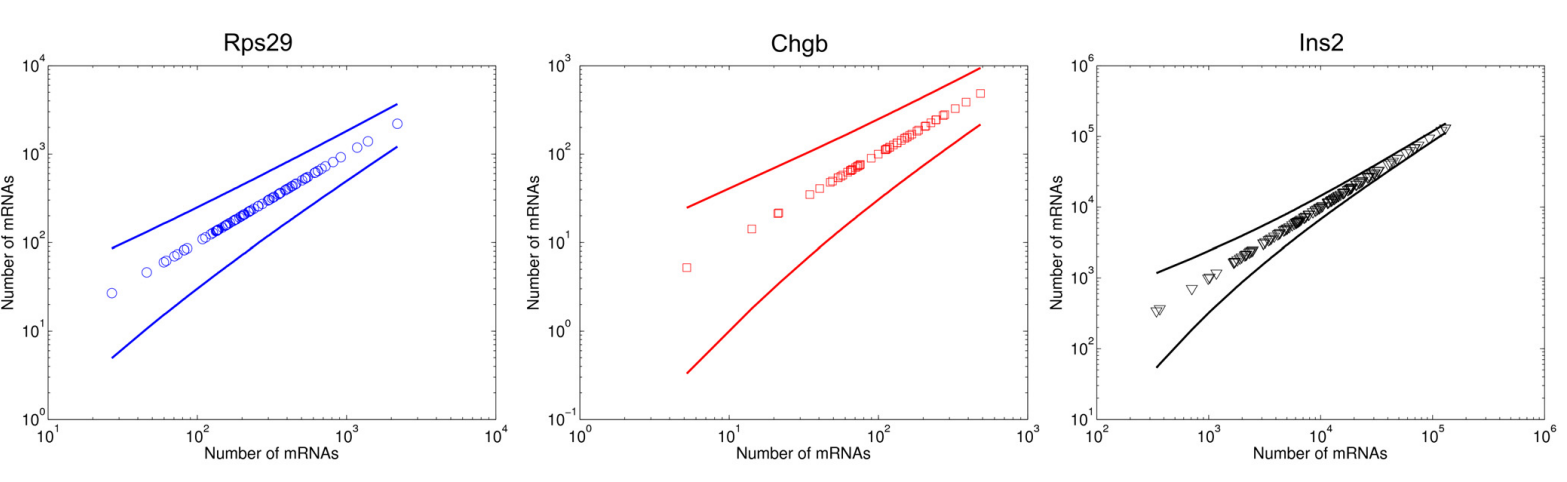
**Confidence intervals for the measurements for *Rps29, Chgb *and *Ins2*.** Data measurements for *Rps29 *(left), *Chgb *(centre), and *Ins2 *(right) with lines showing upper and lower 95% confidence intervals for different amounts of mRNA based on a log-normal distribution with parameters estimated from the experimental data.

How much of this variability stems from true, biological variation and how much is attributable to the RT-qPCR? The RT-qPCR noise model applied on the single-cell data suggests that the RT-qPCR noise is ~0.1% of the total variation in the *Ins2 *expression (Figure 4). For *Rps29*, which has lower expression, the range is 10–50% and for *Chgb *75–200%. Thus, the decreasing accuracy of the RT-qPCR at lower copy numbers heavily influences measurements of low-abundance transcripts such as *Chgb*. Moreover, the 95% confidence level shows that a single measurement is accurate to within a factor of two when the expression level is >1000 transcripts (Figure 5). We conclude that in the case of abundantly expressed genes, the distribution of the measurement errors is very narrow compared to the biological noise and the fluctuations introduced by the experimental procedure are negligible.

Finally, we investigated PCR inhibition caused by overloading the reaction with cDNA [11,22-24]. We tried to mitigate the PCR inhibition by decreasing the ratio between reverse transcriptase and Taq polymerase, and found that a ratio ≤ 2 (U/U) was required to completely remove inhibitory effects. At low concentrations of RNA, decreasing the RT-enzyme concentration did not have a negative effect on RT-yield (Additional file 1: Table 2).

## Discussion

Individual cells have transcript levels which vary significantly from the population mean [1-6,14,19]. The biological significance of this heterogeneity is still not clearly understood and it is an active area of research today. To characterize this cell-to-cell variation, high precision measurements of single cells are required. Technically, such measurements are still very challenging and we have presented an RT-qPCR protocol which should be accessible for most laboratories.

By using a purification-free protocol we are both simplifying single-cell measurements and eliminating RNA losses associated with current available purification protocols. High concentrations (≥ 4 M) of guanidine salts are commonly used in standard RNA purification protocols, where it denatures nucleases rapidly and liberates nucleic acids from bound proteins [25-29]. Since GuSCN strongly inhibits downstream reactions, the RNA is normally purified. At lower concentrations of GuSCN the effect diminishes, but RNases are completely inhibited even at 1 M GuSCN [30] and we show that the cell lysis ability remains strong at 0.5 M. By ensuring sufficient dilution (10-fold) of the GuSCN prior reverse transcription, the concentration is brought down to regions where it does not interfere with the enzymatic reactions. Indeed, we demonstrate that low concentrations (~40 mM) even had a *favourable *effect on the RT yield and reproducibility. This effect may be a result from reduced secondary structures of the mRNA [25], enhanced enzyme activity [31], and/or that GuSCN protects the mRNA from degradation.

We have also developed a mathematical model which allows us to quantify the contributions from the RT and PCR steps to the technical noise. The only parameter in our model which is not directly obtained from the experimental data is the RT efficiency. The RT efficiency can be estimated experimentally by RT-qPCR using known amounts of synthetic RNA mimicking the properties of the native mRNA [21]. However, producing full length mRNAs is challenging and it is common to use a truncated RNA molecule which may not be reverse transcribed with the same efficiency as the native mRNA. Instead, we use our model to determine the RT efficiency indirectly; using non-linear regression we find the RT efficiency which best fits the data. We find that this indirect method gives realistic results for *Chgb*, *Rps29, Gfap*, Nes and *Sox2 *and we believe that it could be a useful way of estimating the RT efficiency for a given gene.

**Figure 6:**
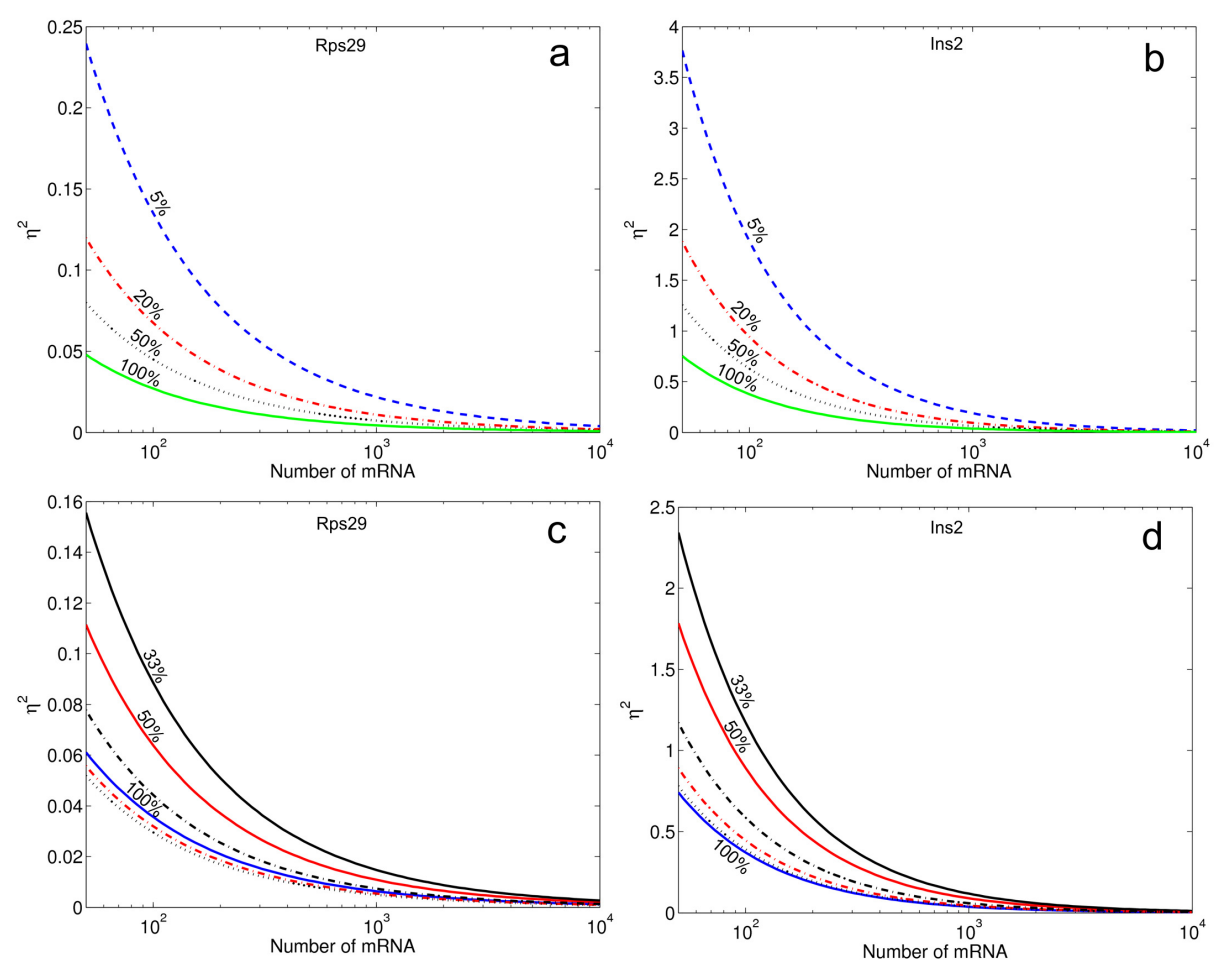
**Effect of cDNA load and replicate qPCR on total noise levels for *Rps29 *(left, a and c) and *Ins2 *(right, b and d).** Using our mathematical model, we evaluate different ways of splitting a sample before measurements. Panels (a) and (b) show how the technical noise is affected by splitting the cDNA 2-(dotted black line), 5- (dash-dotted red line) and 20-times (dashed blue line) before the qPCR. The solid green line represents the case when all cDNA generated is analyzed in one single qPCR measurement. The percentages indicate the fraction of the cDNA that is loaded to the qPCR. Panels (c) and (d) show technical noise levels as a function of initial mRNA copy number when either all cDNA (100%, solid blue line), half of the cDNA (50%, solid red line), or a third of the cDNA (33%, solid black line) is measured in one single qPCR. The dashed lines shows the combined noise levels from duplicate qPCR reactions from the cDNA split in two (red) and three (black) parts. Finally, the black dotted line shows the technical noise when the cDNA is analyzed with triplicate qPCR reactions.

Given the limited amount of mRNA in a single cell [32], how should the RT and qPCR experiments be designed in order to minimize technical noise? We find that the benefit of replicate RT reactions is insignificant, depending on the RT efficiency, and we recommend a single RT reaction using all available mRNA (Additional file 2). In Figure 6, we illustrate different strategies to distribute the cDNA in qPCR reactions. High dilution allows more genes and replicates to be analyzed, but this is at the price of lower mRNA copy numbers, thereby increasing the measurement noise. Figures 6a–b shows how the technical noise increases when the sample is diluted. Curves show technical noise levels for each sample when it is split in 1, 2, 5 or 20 equal parts between RT and PCR. Figures 6c–d show that additional dilution of the cDNA to allow qPCR replicates is not a suitable approach to decrease the technical noise. In particular, for *Ins2 *(Figure 6b), which has a low RT efficiency there is a slight increase of the technical noise when the number of replicates is increased. In contrast, for *Rps29 *with a high RT efficiency there is a slight reduction of the technical noise for low mRNA numbers.

In summary, the single-cell mRNA should be reverse transcribed in a single RT reaction followed by one qPCR reaction per gene. It is advantageous to use more of the single-cell cDNA for low-abundance genes and a smaller part for abundantly expressed genes. These conclusions are only valid for low mRNA levels. However, loading large volumes of cDNA to the qPCR may cause inhibition. In an effort to reduce this effect, we confirm previous findings suggesting a lowered reverse transcriptase concentration and an increased Taq enzyme concentration [22,23]. An alternate solution is to precipitate the single-cell cDNA in ethanol, thus utilizing a larger fraction of the total amount in the qPCR [11]. Yet another option is *multiplex *pre-amplification RT-PCR for 10–15 cycles, followed by singleplex qPCR on the resulting pool of PCR-products[33,34]. This could decrease technical noise levels, provided that the multiplex PCR is efficient, reproducible and unbiased.

Our model of the technical noise suggests that most of the noise in the method stems from the qPCR, at least when the initial template copy number is low and the RT-efficiency is relatively high. However, our analyses show that the RT-efficiencies vary significantly across genes [21,35] and for low efficiencies, the RT reaction will constitute a larger proportion of the total noise. Compared to the biological cell-to-cell variation, the technical noise is insignificant down to ~1000 copies, but becomes considerable at <100 RNA copies. The model includes only three experimental factors; the RT-reaction, dilution of the sample and the qPCR while in reality there are many more steps contributing to the total noise. Thus, our estimates of the noise components should be considered upper bounds since they will most likely become smaller if additional factors are included in the model.

## Conclusion

We found that the strong denaturant guanidine thiocyanate can serve as lysis agent and RNase inhibitor and improve reverse transcription yield in purification-free single-cell RT-qPCR. Our protocol allows fast and streamlined measurements of single-cell gene expression. Analysis of technical noise caused by the RT- and PCR-reaction showed that this noise is insignificant compared to biological cell-to-cell variation at mRNA copy numbers >~100. Below this level technical noise stemming from the PCR, which increases dramatically when initial copy number is <~20 cDNA copies, becomes significant. In addition, to achieve a high technical reproducibility, genes should be analyzed with lowest possible dilution between the RT and qPCR. That is, a single qPCR measurement should be performed for each gene, where the number of genes to be analyzed determines the total dilution factor between RT and PCR.

## Methods

### Preparation and culture of cells

Pancreatic islets were prepared from healthy female National Maritime Research Institute (NMRI) mice aged 3–4 months (Bomholtgaard) and fed a normal diet ad libitum. The mice were sacrificed by cervical dislocation, and pancreatic islets were isolated by collagenase P digestion (Roche) followed by manual collection of islets [36]. To prepare dispersed single cells the collected islets were gently shaken at low extracellular Ca^2+ ^concentration to dissolve the structure of the islet [37]. Dispersed cells were plated in plastic 35 mm Petri dishes (Nunc) in RPMI 1640 medium (SVA) supplemented with 10% FCS, 100 U ml^-1 ^penicillin, and 10 μgml^-1 ^streptomycin (all from Invitrogen) in the presence of 5 mM glucose (Sigma-Aldrich). The cells were maintained in culture for 2–6 hours, allowing them to attach to the surface of the dish

Primary astrocytes were prepared and cultured as described [38]. Cells were grown in Dulbecco's modified Eagle's medium (Sigma-Aldrich) containing 10% fetal calf serum, 2 mmol/L L-glutamine, and penicillin-streptomycin (all Invitrogen).

### Single cell collection

Dispersed attached cells were washed twice with a buffer (referred to as extracellular solution, EC) containing 138 mM NaCl, 5.6 mM KCl, 1.2 mM MgCl_2_, 2.6 mM CaCl_2_, 5 mM HEPES (pH 7.4 with NaOH) and 3, 6, 10 or 20 mM glucose to remove dead and loose debris prior to cell collection with patch-clamp pipettes. The dish, containing adhered cells and approximately 1 ml EC-solution, was mounted in a standard inverted light-microscope (Zeiss Axiovert 135). Borosilicate glass capillaries (Hilgenberg GmbH) with outer diameter of 1.5–1.6 mm and wall thickness of 0.16 mm were pulled to pipettes using a patch-clamp pipette puller (Heka PIP5). The diameter of the tip was approximately 10 μm on average, substantially wider than standard patch-clamp pipettes and large enough to allow passage of an intact cell. The glass pipette was mounted on a hydraulic micromanipulator (Narishige) on the microscope. By manually controlling the pressure inside the pipette it was possible to collect intact or nearly intact cells with minimum volume of extracellular solution (<<0.1 μl). In total, 158 cells were collected, and 126 (80%) were identified as β-cells based on their expression of insulin while most of the remaining cells were α-cells (16%). Six samples were negative for all measured genes and they were categorized as technical failures (96% success rate).

### Lysis and purification

#### Cell-cluster lysis

Single pancreatic islets of roughly the same size were placed in 10 μl of various lysis buffers. The detergents Nonidet P-40 (NP-40, also known as Igepal CA-630, Sigma-Aldrich) and guanidine thiocyanate (GuSCN, Sigma-Aldrich) were used. Samples were incubated at 60°C or 80°C for 15 minutes (60°C for samples containing 0.4 mg/ml proteinase K (Invitrogen)) followed by 5 min incubation at 95°C and frozen at -25°C for subsequent analysis. Samples were diluted 1:20 with mQ water (ELGA Labwater) prior reverse transcription.

Primary astrocytes were washed twice in PBS followed by single cell dissociation with 0.25% Trypsin/EDTA (Invitrogen) treatment for 3 min. Trypsin was inactivated by addition of cell medium and washed once in PBS. Cells were then filtrated using a 40 μm cell strainer (Becton Dickinson). Cells were evenly distributed between sample tubes and all liquid were eliminated after a short centrifugation (3000 g for 1 min) followed by addition of 50 μl lysis buffer. 2 U/μl RNaseOut (Invitrogen) was used as RNase inhibitor. Samples were incubated at 80°C for 5 minutes and frozen at -25°C for subsequent analysis. Samples were diluted 1:4 with mQ water prior reverse transcription.

#### Single-cell lysis

In single-cell experiments, the glass pipettes were emptied in 0.2 ml plastic tubes containing either 2 μl of 0.5 M GuSCN or 1 μl of 1 M GuSCN in water. The emptying required a custom-made device consisting of a tube holder lined up with a coarse micromanipulator on which the pipette was mounted. The glass pipette was carefully flushed with lysis solution a few times to make sure the cell entered the tube. In most cases, the tip of the pipette was gently broken in the tube thereby facilitating the flushing of the pipette. Tubes were then immediately frozen on dry ice (-78°C) and stored at -80°C for subsequent reverse transcription. Short-term storage on wet ice (0°C) did not affect degradation or subsequence reaction performance.

#### Total RNA extraction

Total RNA was purified with GenElute Mammalian Total RNA Kit (Sigma-Aldrich) or RNeasy Mini Kit (Qiagen) and concentrations were measured with a NanoDrop ND-1000 spectrophotometer (Nanodrop Technologies).

### In vitro transcription

An artificial RNA control was generated with the T7 RNA Polymerase in vitro transcription system (Takara). A PCR assay for cyclophilin E (*Ppie*) was used as template for the in vitro transcription. *Ppie *PCR product was generated using the protocol as for the SYBR Green-based real-time PCR assays (see below), except that all fluorophores were excluded, and purified using QIAquick PCR purification kit (Qiagen). The PCR product was then amplified using an extended forward PCR primer including the promoter sequence for T7 RNA Polymerase. All primer sequences are found in Additional file 1: Table 3. The resulting (extended) PCR product was purified as above and used in the in vitro transcription reaction, according to the manufacturer's instruction. The reaction mixture contained: 50 U T7 RNA polymerase, 40 mM Tris-HCl (pH 8.0), 8 mM MgCl_2_, 2 mM spermidine, 5 mM dithiothreitol (all Takara), 2 mM NTP (Invitrogen), 20 U RNaseOut (Invitrogen) and ~40 ng template DNA. The reaction was carried out at 42°C for 1 h.). In samples containing ~2000 copies of the *Ppie *RNA, the average intra-assay coefficient of variation was 0.3% and the inter-assay variation was 1.2%.

### Quantitative RT-PCR

Due to stochastic fluctuations, *relative *gene expression levels provide little information, leaving absolute quantification using standard curves as the best option to compare transcript levels within and between cells. For all assays, PCR-products were purified (QIAquick PCR purification kit, Qiagen) and quantified spectrophotometrically (NanoDrop ND-1000) using the following molar absorptivity values (in Moles^-1^cm^-1^): dAMP, 15200; dTMP, 8400; dGMP, 12010; dCMP, 7050. The PCR-products were diluted in series to generate a standard curve ranging 10-10^6 ^copies with PCR-efficiencies spanning 77–95% (Additional file 1: Figure 3). qPCR data analysis was performed essentially as described [9]. Since PCR-products are double-stranded and cDNA is single-stranded, one cycle was subtracted from single-cell Ct-values for correct absolute quantification.

The reverse transcriptase SuperScript III (Invitrogen) was used throughout the study. 6.5 μl containing total RNA or lysed single cells, 0.5 mM dNTP (Sigma-Aldrich), 2.5 μM oligo(dT) (15 bp, Invitrogen), 2.5 μM random hexamers (Invitrogen) were incubated at 65°C for 5 min. We then added 50 mM Tris-HCl (pH 8.3), 75 mM KCl, 3 mM MgCl_2_, 5 mM dithiothreitol, 20 U RNaseOut and 100, 40 or 10 U (lower amount for single-cell-samples) SuperScript III (all Invitrogen) to total volume of 10 μl. Final reaction concentrations are shown. The temperature profiles used were 25°C for 5 min, 37°C for 10 min, 50°C for 80 min. All reactions were terminated at 70°C for 15 min. Throughout this paper we refer to absolute copy numbers estimated by DNA-based standard curves; assuming an RT efficiency of ≤ 100%, the true number of mRNA copies is equal to or higher than our estimate.

ABI PRISM 7900 HT Sequence Detection System (Applied Biosystems) and LightCycler 480 (Roche Diagnostics) equipped with 384-well blocks were used for qPCR measurements. Reactions (10 μl) with SYBR Green as fluorescent reporter contained 10 mM Tris (pH 8.3), 50 mM KCl, 3 mM MgCl_2_, 0.3 mM dNTP, 1 U JumpStart Taq polymerase, 1 × Reference Dye (all Sigma-Aldrich), 0.5 × SYBR Green I (Invitrogen) and 400 nM of each primer (TAGC Copenhagen). The Reference Dye was excluded using LightCycler 480. The iQ™ SYBR^® ^Green Supermix (BioRad) was used for the *Nes *assay. The primer sequences are available in Additional file 1: Table 3. All qPCR assays were designed using Primer3Plus  and spanned at least one intron when possible. They were optimized to not generate any unspecific products, such as primer-dimers, that could interfere with the quantification of the desired target. Formation of expected PCR products was confirmed by agarose gel electrophoresis (2%) for all assays, and melting curve analysis for all samples.

For RT-qPCR noise calculations, 24-plicate RT-qPCR on diluted purified total mouse islet RNA was used and 30-plicate qPCR reactions on total islet cDNA.

### Analysis of RT-qPCR noise

The theoretical model is based on an empirical characterization of the noise in the experiments. First, we consider the noise in the qPCR reaction only and determine how it scales as a function of the number of cDNAs based on replicates which have been diluted to different degrees. The qPCR noise is assumed to be the same for all genes. To model the RT-noise a parameter for the RT efficiency is fitted from replicates which have been diluted to different degrees. Using our empirically determined parameters for the model, we can calculate the contributions of the RT and qPCRs and the total measurement noise can be compared to the biological variation in a population of cells. A full description of the model is given in Additional file 2.

## Abbreviations

GuSCN: guanidine thiocyanate; RT-qPCR: quantitative reverse transcription PCR.

## Authors' contributions

MB and AS conceived and designed the study, and carried out all experiments. MB coordinated the study and drafted the manuscript. MH performed the data analysis of all single-cell measurements and developed the noise model. PR helped draft the manuscript. All authors participated in the writing of the manuscript and approved the final version.

## Supplementary Material

Additional file 1 containing Figures 1, 2, 3, 4 and Tables 1–3. Click here for file

Additional file 2 containing a detailed description of the mathematical model. Click here for file
