# Supplementary material for: Quantification of mRNA in single cells and modelling of RT-qPCR induced noise
Source: BMC Mol Biol. 2008 Jul 17;9:63. doi: 10.1186/1471-2199-9-63 (PMC2483285; doi:10.1186/1471-2199-9-63)
Supplement: Additional file 1 — containing Figures 1, 2, 3, 4 and Tables 1–3. [file 1471-2199-9-63-S1.pdf]

<u>Additional file 1:</u> Supplementary figures and tables for

Quantification of mRNA in single cells and modelling of RT-qPCR induced noise

Martin Bengtsson, Martin Hemberg, Patrik Rorsman and Anders Ståhlberg

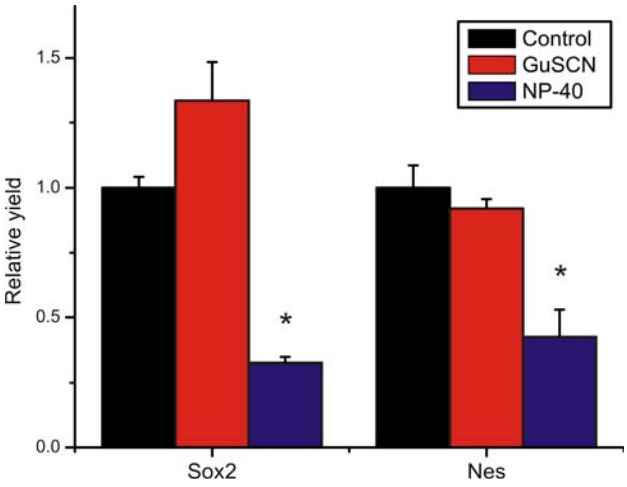

Additional File 1, Figure 1: Evaluation of lysis buffers on primary astrocytes. Primary astrocytes were dissociated into single cells by Trypsin/EDTA treatment. Trypsin was inactivated by cell medium and washed once in PBS. Each bar indicates relative yield of *Sox2* and *Nes* from equally pooled astrocytes as starting material. Each sample was treated with indicated concentrations of either mQ-water (control), 0.5 M guanidine thiocyanate (GuSCN) or 0.5% NP-40 all supplemented with RNase inhibitor (2U/µL). The value of the control was arbitrarily set to 1. Values are mean ± SEM for 4 separate experiments (* $p < 0.01$ compared to control).

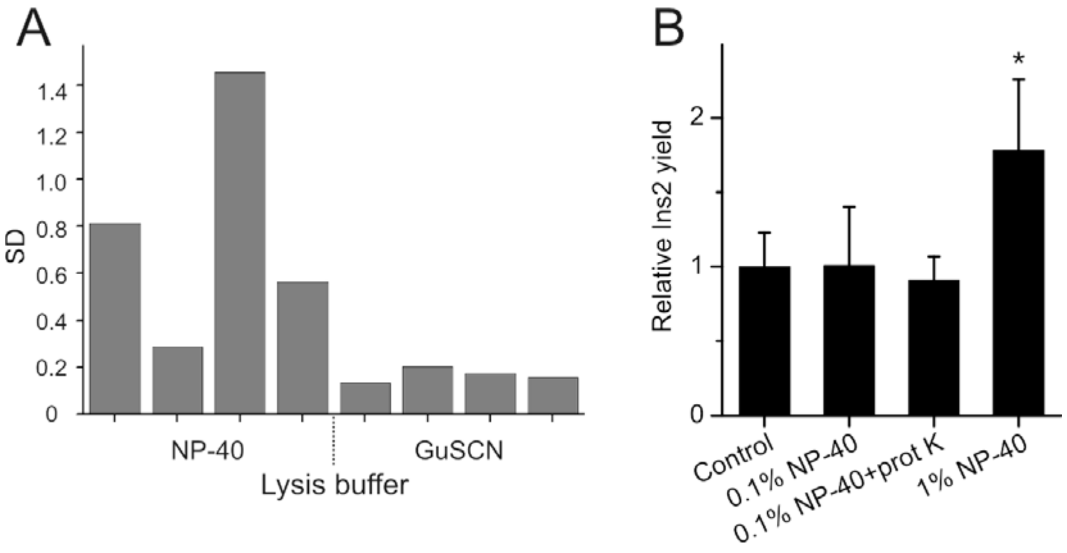

Additional file 1, Figure 2: (A) Eight single-cell samples analysed in triplicate RT-reactions; four samples with lysis buffer containing 0.5% NP-40 and four with 0.5 M GuSCN. Intra-assay variation for each sample is shown. (B) Effect of lysis buffers on RT reaction yield. Identical amounts of purified islet total RNA were used as starting material. Relative yield of reverse transcribed *Ins2* are shown for samples supplemented with NP-40 and proteinase K (prot K). The value of the control (water) was arbitrarily set to 1. Values are mean ± SEM for 3 separate experiments (* $p < 0.01$ compared to control).

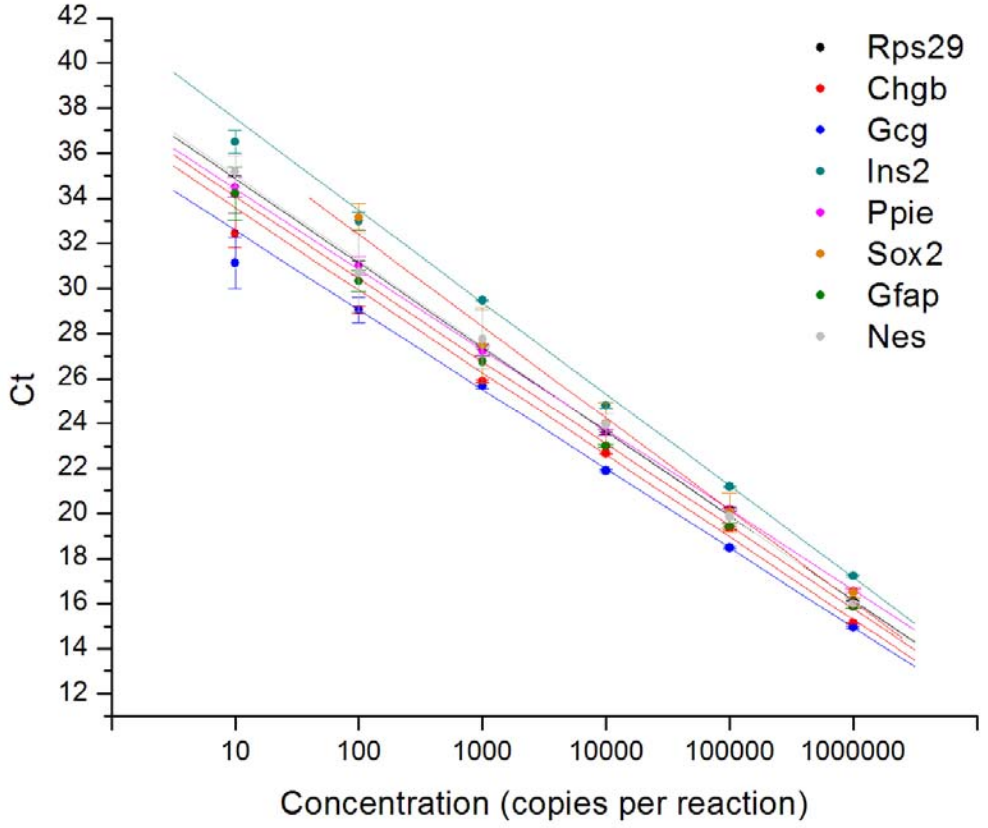

Additional file 1, Figure 3: Standard curves based on purified PCR-products. Each data point is the average of triplicate reactions with SD indicated with bars. The standard curve is generated by linear regression (R > 0.99 for all assays). The slope (a) is used to derive the PCR efficiencies, using the equation $E = 10^{(-1/a)} - 1$ (*Rps29*: 0.86; *Chgb*: 0.95; *Gcg*: 0.92; *Ins2*: 0.81; *Ppie*: 0.90; *Sox2*: 0.77; *Gfap*: 0.88; and *Nes*: 0.85).

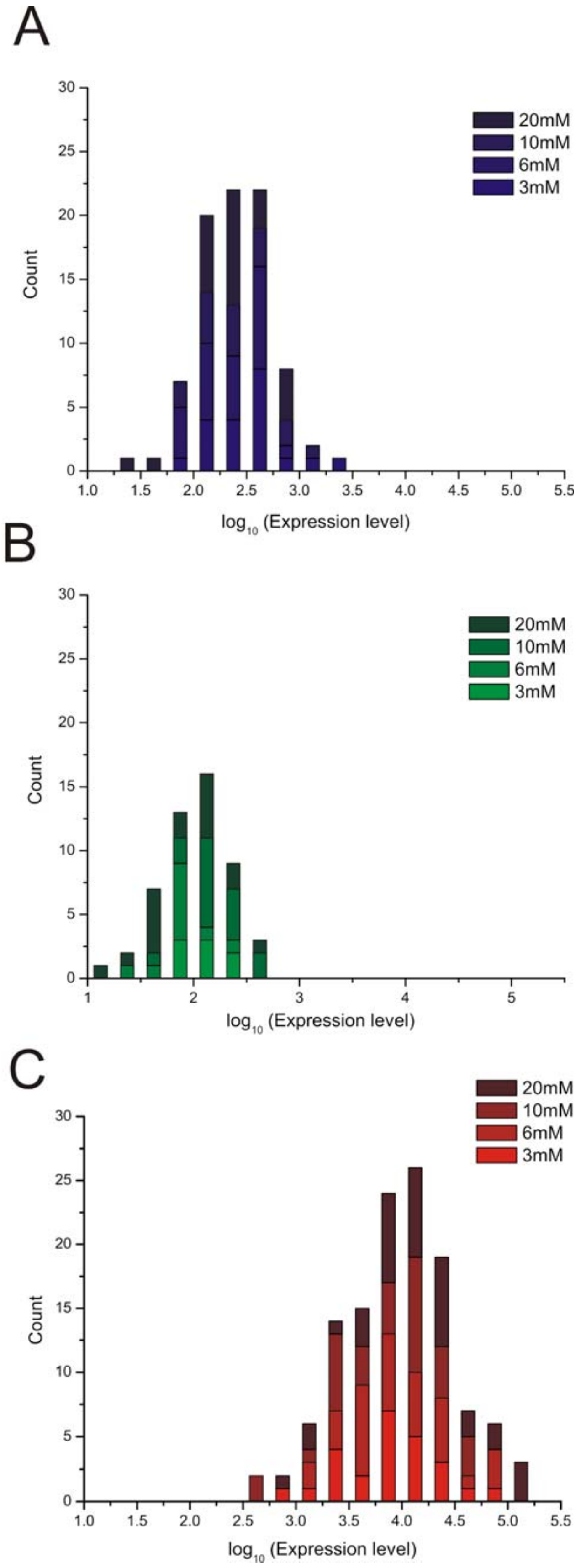

Additional file 1, Figure 4: Histograms showing expression levels of *Rps29* (A), *Chgb* (B) and *Ins2* (C) in single pancreatic β-cells at four different glucose concentrations (3, 6, 10 and 20 mM).

Additional file 1, Table 1: Effect of contaminants from cell collection on downstream reaction yields

Gene	Control	1) EC	2) one cell	3) ten cells
Ins2	1.0 (0.15)	0.98 (0.21)	1.9* (0.67)	2.4* (1.1)
Gcg	1.0 (0.20)	1.1 (0.21)	2.2* (0.71)	2.9* (1.4)
Hprt	1.0 (0.18)	1.0 (0.24)	1.8* (0.20)	1.5 (0.30)

Fold difference (SD) in qRT-PCR yield compared to control conditions (water) when complementing the reverse transcription reaction with: 1) 0.2 µl extracellular solution (EC); 2) one or 3) ten cell/s collected in EC and transferred to 1 µl lysis buffer containing 0.5 M GuSCN. All tubes were complemented with identical amounts of purified total islet RNA prior the reverse transcription reaction. * $P<0.05$ compared to corresponding control (n=8).

Additional file 1, Table 2: Mitigation of PCR-inhibition by reduction of RT-enzyme concentration and increase in Taq polymerase-concentration

cDNA load into PCR:	5% cDNA	20% cDNA	20% cDNA
Enzyme units (U) per reaction:	0.25 U Taq	0.25 U Taq	2.75 U Taq
100 U RT	*21.0 (20)*	*No signal (80)*	*24.5 (7.3)*
40 U RT	*20.1 (8)*	*No signal (32)*	*22.1 (2.9)*
10 U RT	17.2 (2)	*19.7 (8)*	15.6 (0.7)

Average Ct-values of Ins2 and (in parenthesis) RT:Taq ratio (U/U) in the PCR-reaction. Italic style indicates inhibited PCR. Both RT- and PCR-reactions were 10 µl. Taq = Taq polymerare, RT = reverse transcriptase.

Additional file 1, Table 3: PCR primer sequences

Gene abbrev.	Full name	Fwd primer (5'-3')	Rev primer (5'-3')	Intron spanning	NCBI Gene ID
Ins1	Insulin I	TAGTGACCAGCTATAATCAGAG	ACGCCAAGGTCTGAAGGTCC	yes	16333
Ins2	Insulin II	CCCTGCTGGCCCTGCTCTT	AGGTCTGAAGGTCACCTGCT	yes	16334
Gcg	Glugacon	CTACACCTGTTCGCAGCTCA	CTGGGGTTCTCCTCTGTGTC	yes	14526
Rps29	Ribosomal protein S29	GGAGTCACCCACGGAAGT	TCCATTCAAGGTCGCTTAGTC	yes	20090
Chgb	Chromogranin B	CAAATGCCCTATCCAAGTCC	TCACCTTTGACCTCTTTTCCA	yes	12653
Hprt	hypoxanthine guanine phosphoribosyl transferase	AGCCCCAAAATGGTTAAGGT	CAAGGGCATATCCAACAACA	yes	15452
Sst	Somatostatin	CCACCGGGAAACAGGAAC	GCTCCAGCCTCATCTCGTC	yes	20604
Gapdh	Glyceraldehyde-3-phosphate dehydrogenase	AGCGAGACCCCACTAACATC	GGTTCACACCCATCACAAAC	yes	407972
Sox2	SRY-box containing gene 2	AAAAACCACCAATCCCATCC	AGTCCCCCAAAAAGAAGTCC	no[1]	20674
Gfap	Glial fibrillary acidic protein	AACCGCATCACCATTCCT	CGCATCTCCACAGTCTTTACC	yes	14580
Nes	Nestin	AGCAACTGGCACACCTCAA	GGTATTAGGCAAGGGGGAAG	yes	18008
Ppie	Cyclophilin E (control)	CAACGGCTCCCAGTTCTTCC	TCTGCTTTGGCTTCCCATCC	N.A.	56031
Ppie	Extended forward primer including T7 promoter	ATTAATACGACTCACTATAGGG AGACAACGGCTCCCAGTTCTTCC			

[1]single-exon gene; intron-spanning primer design not possible. N.A. = not applicable.
