# Supplementary material for: Quantification of mRNA in single cells and modelling of RT-qPCR induced noise
Source: BMC Mol Biol. 2008 Jul 17;9:63. doi: 10.1186/1471-2199-9-63 (PMC2483285; doi:10.1186/1471-2199-9-63)
Supplement: Additional file 2 — containing a detailed description of the mathematical model. [file 1471-2199-9-63-S2.pdf]

Additional file 2: Mathematical Models of RT-qPCR Noise

Martin Bengtsson Martin Hemberg Patrik Rorsman Anders Ståhlberg

July 14, 2008

Mathematical Model

Here a phenomenological model of the noise in the RT-qPCR is presented. The basis of the model is the identification of independent components contributing to the noise. The contributions of the different steps involved in the procedure are estimated and quantified by fitting the model parameters from the data.

Noise in the PCR

First, the noise for the PCR only was analyzed based on the data from 30 replicate measurements of the same sample at five different concentrations. For this reaction it is assumed that there are two noise sources, stemming from the dilution relative to a reference sample and the PCR respectively. Since the dilution and the PCR occur in succession, they are assumed to be independent. A full description would account for the fact that the noise stemming from the dilution may propagate to the PCR, but as a first approximation, these effects are neglected. Moreover, this model overestimates the noise in the PCR since it neglects the noise from all the other steps in the protocol.

The noise strength, or normalized variance $\eta^2 = \sigma^2/\mu^2$, is used to quantify the noise level. The assumption that the dilution and PCR noises are independent allows the total noise η^2_{DP} to be written as

$$\eta^2_{DP} = \eta^2_D + \eta^2_P, \tag{1}$$

where η^2_D is the dilution noise and η^2_P is the PCR noise.

The dilution process is modelled as a random process with a binomial distribution, $N_D \sim \text{Bin}(p, N)$, where the parameter p is equal to $1/D$ if the sample was diluted 1:D, N is the number of DNA template molecules prior to the dilution and N_D is the number of DNA molecules after the dilution. The rationale for this choice of distribution is that when the initial substrate is diluted, each template has a probability $1/D$ of ending up in the sample being analyzed. The mean and variance of a binomial random variable are given by Np and $Np(1-p)$, respectively. Thus, the dilution noise becomes

$$\eta^2_D = \frac{1-p}{Np} = \frac{D-1}{N}. \tag{2}$$

The PCR noise depends on the number of cycles and the data shows the number of cycles which has to be run to reach a set fluorescence level or cycle threshold (Ct). Figure 1 confirms that Ct is very close to a normal distribution. Since the number of cDNAs is given by $N = k(1+\beta)^{Ct_0 - Ct}$, it follows that N will have a log-normal distribution. Here β is the PCR efficiency, k is a proportionality constant and Ct_0 is a constant determined from a calibration curve and it corresponds to the Ct-value of a single DNA template molecule in the initial sample.

Figure 2 shows how the noise for the PCRs decay as the number of molecules in the initial sample increases. In the log-log scale, the relation becomes linear and the PCR noise can be written as

$$\eta^2_P = \exp(a + b \log N/D). \tag{3}$$

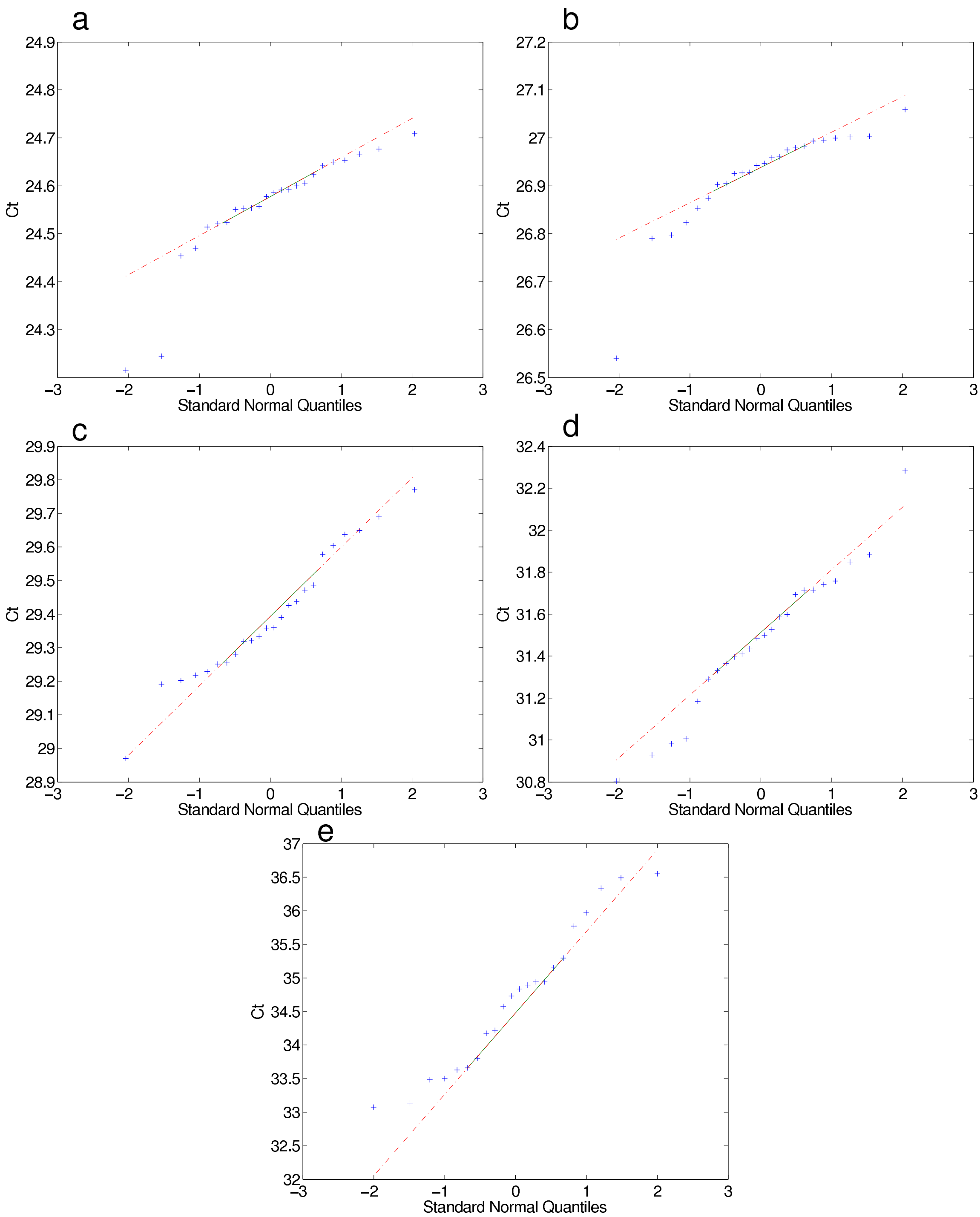

Figure 1: **Quantile-quantile plots for the number of cycles in the qPCRs for different dilutions.** (a-e) $D = 1, 4, 8, 16, 64$ respectively. The pluses represent the data and the lines correspond to normal distributions. In all cases, the normality assumption appears to hold well. In total, there were only three outliers which were removed from the subsequent analysis, two are found in (a) and one in (b).

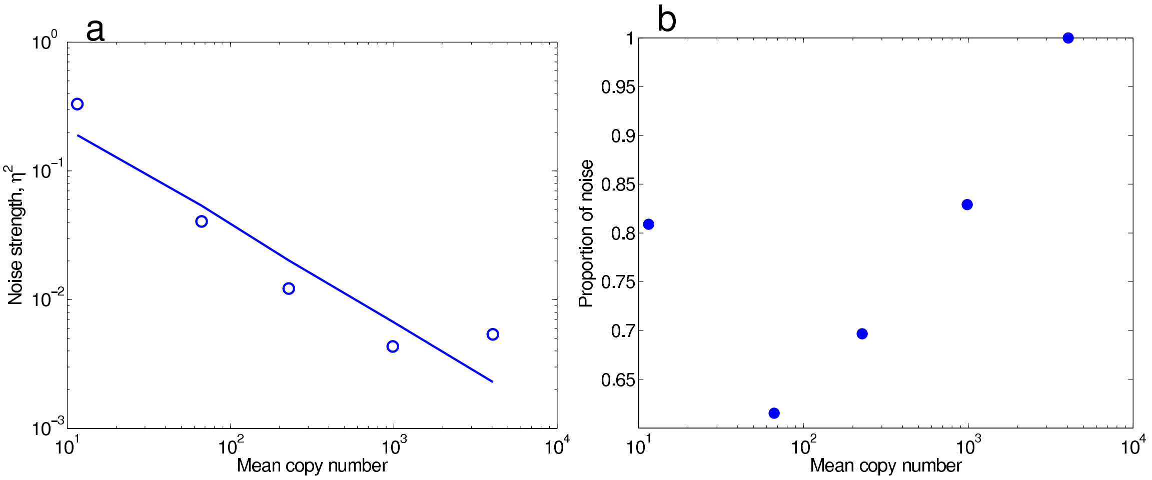

Figure 2: **Noise in the PCR.** (*a*) The total noise as a function of the number of DNA template molecules at the start of the reaction. The circles correspond to the measured noise levels and the line is the fit provided by Eq (1)-(3). (*b*) The PCR noise corresponds to approximately 75% of the total noise for the samples which have been diluted with respect to a reference sample. The reference corresponds to the rightmost point by construction the noise is considered to stem from the PCR only.

From the data in Figure 2, the parameters are found as $a = -0.39$ and $b = -0.68$. The dilution noise in Eq. 2 decreases as N^{-1} which means that the PCR noise decreases slower and consequently it will come to dominate for large N.

Noise in the RT-PCR

When the RT step is included, an additional term, η_R^2, stemming from the reverse transcription which takes place after the dilution and before the PCR step is added

$$\eta_{DRP}^2 = \eta_D^2 + \eta_R^2 + \eta_{D'}^2 + \eta_P^2. \tag{4}$$

In the experiment, the RT-reaction was followed by a second dilution before the sample underwent PCR which is accounted for by the term $\eta_{D'}^2$. For *Rps29*, *ChgB* and *Ins2*, this factor is 5 and for *Gfap*, *Nes* and *Sox2*, the factor is 10. This means that the molecule numbers prior to this dilution should be multiplied by a factor of D'.

It is assumed that the RT-reaction can be modelled as a binomial process with parameters α and $D'N/D$, where α is the efficency of the RT-reaction and $D'N/D$ is the expected number of mRNAs follwing the dilution. The motivation for this choice of distribution is that under the assumption that there is an excess amount of enzymes present, each mRNA molecule will have a probability α of undergoing the RT reaction. Thus, the RT reaction can be viewed as flipping a coin with probability α for each transcript present in the original sample. As will be shown later, the RT efficiency may differ from gene to gene. For the dilution and the RT-reaction the propagation of the noise can be taken into account. Due to the relatively simple form of the two processes, Burgess variance theorem [1] can be applied to account for the noise propagated from the dilution to the RT-reaction

$$\sigma_R^2 = \alpha^2\sigma_D^2 + \alpha(1-\alpha)D'N/D = \frac{\alpha^2 D'N(D-1)}{D^2} + \frac{\alpha(1-\alpha)D'N}{D},$$

and it follows that the noise strength becomes

$$\eta_R^2 = \alpha^2\eta_D^2 + \frac{\sigma_{DR}^2}{(D'N\alpha/D)^2} \quad \Rightarrow \quad \eta_{DR}^2 = \eta_D^2 + \eta_R^2 = (1+\alpha^2)\frac{D-1}{D'N} + \frac{(1-\alpha)D}{D'\alpha N}. \tag{5}$$

The two components correspond to the dilution and the RT-process, respectively. Applying the Burgess variance theorem again, the noise from the RT and the two dilutions becomes

$$\eta^2_{DRD'} = \frac{(D'^2+1)(1+\alpha^2)}{D'^2}\frac{D-1}{D'N} + \frac{(D'^2+1)}{D'^2}\frac{(1-\alpha)D}{D'\alpha N} + \frac{(D'-1)D}{D'\alpha N}. \tag{6}$$

For the PCR, it is assumed that the noise-characteristics are the same as in the previous section with PCR only. An important difference from before is that the RT-efficiency must be taken into consideration when calculating the PCR noise. This means that Eq. (3) becomes

$$\eta^2_P = \exp(a + b\log N/\alpha D) \tag{7}$$

Equation (5)-(7) have introduced the RT-efficiency, α, which cannot be obtained directly from these experiments. However, all other quantities in Eq. (4) are known which makes it possible to obtain α by non-linear regression. The fitting problem can be formulated as finding the value of α which gives the best fit for

$$\eta^2 = \frac{(D'^2+1)(1+\alpha^2)}{D'}\frac{D-1}{D'N} + \frac{(D'^2+1)}{D'}\frac{(1-\alpha)D}{D'\alpha N} + \frac{(D'-1)D}{D'\alpha N} + e^{a+b\log(N/\alpha D)} \tag{8}$$

From the experiments, N and D are known and the parameters a and b were estimated in the previous Section. Using Matlab's `lsqcurvefit` command, $\alpha = .85, .67, .03, .99, .28$ and $.08$ are obtained for *Rps29*, *ChgB*, *Ins2*, *Gfap*, *Nes* and *Sox2*, respectively. Once α has been computed, the model can be used to calculate the contribution to the total noise from the three components as shown in Fig. 3*a* in the Main text. An interesting feature of this figure is that the noise deacays as a power-law with exponent -.8 for low copy-numbers. At $N \approx 1000$ the noise drops below .01 which is approximately two orders of magnitude lower than the biological noise (see below).

The RT-efficiency for *Ins2* is clearly much lower than one would expect. By considering Fig. 3*a* in the Main text it is possible to understand why this happens. Unlike the other two genes, the slope of the noise is almost flat and there is hardly any change as N increases and with the given set of parameters, a low value of α is required for the expression to be insensitive to N. Comparing with Fig. 2, it can be seen that the PCR noise has only been estimated using up to $\sim 10^3$ molecules. Based on the results for *Ins2*, it can be hypothesized that the curve flattens at this point and obtains a different slope. Under this assumption, a more realistic value for α is obtained, but unfortunately, the current data does not unambiguously support such a model. Another explanation could be that the RT-efficiency varies non-linearly as a function of the mRNA concentration and this is not accounted for by the model [2].

The effect of the RT-efficiency is further investigated in Fig. 3 where it can be concluded that the noise level varies non-linearly with α. The introduction of the RT-step does not change the noise characteristics significantly. The RT-noise has the same scaling as the dilution-noise and consequently, it will decrease as N^{-1}. This is not surprising since the RT-step has the same noise characteristics as the dilution.

Biological noise

Next, the expression levels in islet cells were measured for the three genes in different glucose concentrations. It has been shown that mRNA distributions in eukaryotes have fat tails [3, 4] and the same holds for the data presented in this paper. A useful way of characterizing a distribution is through its moments which are summarized in Figure 4.

To get an idea of the sensitivity of the biological samples due to the measurement errors, a Monte Carlo strategy is employed to repeat the experiment *in silico*. For each cell j in the sample, a random number is drawn from a log-normal distribution with mean given by the measured value N_j and variance calculated from Equation (8) using the inferred α value for the particular gene. From the simulated measurements, a new mean and standard deviation can be calculate for the gene. By repeating the procedure N_{MC} times, standard errors for the means and variances of the gene can be obtained. The procedure is outlined in pseudo-code below

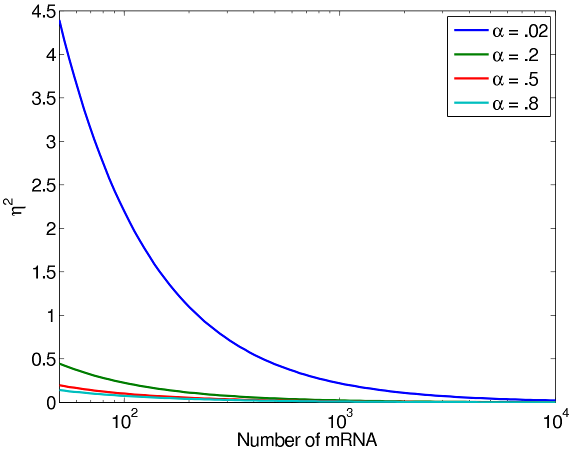

Figure 3: **Noise levels for different values of α.** The noise level varies non-linearly with α and the marginal improvements diminish with increasing RT-efficiency.

---

for i=1:N_{MC} **do**
  for j=1:n **do**
    $\widetilde{\eta}_j^2 \leftarrow$ `noiseStrength`(N_j, D, α)
    $\widetilde{N}_j \leftarrow \text{lognrnd}(N_j, \widetilde{\eta}_j^2 \times N_j^2)$
  end for
  Calculate statistics $\widetilde{\mu}_i$ and $\widetilde{\sigma}_i$ for the samples $\widetilde{N}_j$.
end for
Calculate standard errors for $\widetilde{\mu}$ and $\widetilde{\sigma}$

The function `noiseStrength` calculates the noise strength for a given number of transcripts using Equation (8) and the function `lognrnd` produces a random number from a log-normal distribution with mean and variance given by the two arguments.

From Figure 4*a* it is clear that *Rps29* and *ChgB* are unaffected by the increased glucose concentrations whereas there is a two-fold increase in the expression of *Ins2*. As one would expect, the mean is very robust and even for the *ChgB* gene which has a mean expression level of ~ 100 transcripts, the Monte Carlo simulations show that the standard error as calculated from the simulations is negligble. The variance is more sensitive for the genes with low expression levels. Moreover, it seems as if the variance estimates for these genes are consistently larger than the observed values. This is not surprising since the theoretical model yields noise levels which are larger than the observations for low copy numbers as shown in Fig. 3*a* in the Main text. Figure 4*c* shows that the noise strength is more sensitive to the measurement errors for genes with low expression levels. This is not surprising since η^2 is defined as the ratio between two uncertain quantities. Again, the simulations show higher noise levels which is probably due to the overestimates of the fits for these copy numbers. It is also clear that for *Ins2*, which has a high expression level, the biological noise is three orders of magnitude greater than the noise that one could expect from the measurements as shown in Fig. 4*d*. However, for the two genes with low expression levels, the measurement noise is comparable to the biological noise.

Even though the theoretical analysis yields that the noise of the PCR has a log-normal distribution, it is interesting to note that it has a symmetric distribution which can be equally well fit by a normal distribution. This is in stark contrast to the biological noise which has a long tail to the right [4, 3]. Thus, one can intuitively understand why the measurement noise has a low impact on genes with high expression levels.

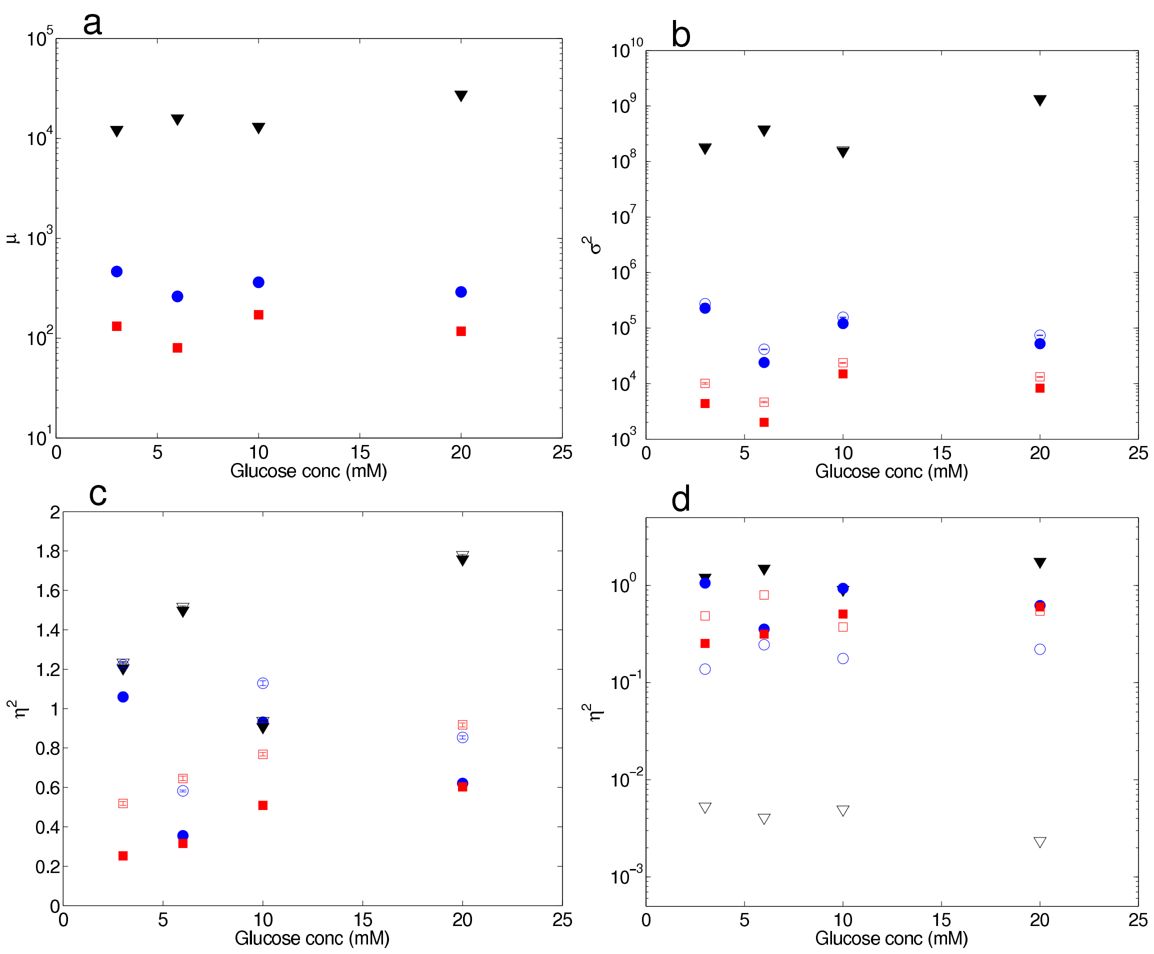

Figure 4: **Summary statistics and fits for the three genes.** (*a*) The mean expression levels for *Rps29* (◯), *ChgB* (□) and *Ins2* (∇) at different glucose concentrations. Closed symbols represent the data and open symbols results from 1000 Monte Carlo simulations with the errorbars given by the standard error. In this panel, there is almost perfect agreement between the data and the simulations and the two symbols are indistinguishable. The first two genes have low expression levels which are unaffected by the glucose concentration. *Ins2* has a high expression level and it is doubled as the glucose is increased from 3 to 20 mM. (*b*) The variance for the data and simulations using the same symbols as in *a*. Here the genes with low expression levels show a larger uncertainty and the discrepancy between the model and the data indicates that the former may not be completely accurate. (*c*) The noise strength for the data and the simulations. Again, a larger discrepancy for the genes with low expression levels is observed. (*d*) The closed symbols denote the noise strength for the biological data as before. The open symbols represent the noise strength of the measurements at the mean expression level. For *Ins2*, there is a difference of three orders of magnitude between the two noise levels which explains the good agreement between the simulations and the measurements. For the other two genes, the difference is much smaller and for *ChgB*, the two noise levels are of the same order of magnitude which indicates that there could be a significant impact from the measurement noise.

References

[1] Burgess RE: **Homophase and Hetrophase Fluctuations in Semiconducting Crystals**. *Disc. Faraday Soc.* 1959, **28**(151).

[2] Ståhlberg A, Håkansson J, Xian X, Semb H, Kubista M: **Properties of the reverse transcription reaction in mRNA quantification**. *Clin. Chem.* 2004, **50**(3):509–515.

[3] Raser JM, O'Shea EK: **Noise in gene expression: origins, consequences and control**. *Science* 2005, **309**:2010–2013.

[4] Bengtsson M, Ståhlberg A, Rorsman P, Kubista M: **Gene expression profiling in single cells from the pancreatic islets of Langerhans reveals lognormal distribution of mRNA levels**. *Genome Research* 2005, **15**:1388–1392.
